# Gamma Radiation in the Synthesis of Inorganic Silica-Based Nanomaterials: A Review

**DOI:** 10.3390/nano15030218

**Published:** 2025-01-29

**Authors:** Andreea Simona Baltac, Raul-Augustin Mitran

**Affiliations:** 1 National Institute for Physics and Nuclear Engineering “Horia Hulubei”, 30 Reactor Str., 077125 Magurele, Romania; andreea.manea@nipne.ro; 2 “Ilie Murgulescu” Institute of Physical Chemistry, Romanian Academy, 202 Splaiul Indepedentei, 060021 Bucharest, Romania

**Keywords:** gamma radiation, radiolytic reduction, silver nanoparticles, porous silica, nanocomposites

## Abstract

Gamma radiation offers a versatile approach for the synthesis of silica-based nanomaterials, leveraging high-energy radiolysis to produce pure and finely structured composites without the need for surfactants or capping agents. This review explores the underlying mechanisms of γ-ray-induced radiolytic reduction, detailing the interaction of radiolytic species with silica matrices to synthesize metallic and hybrid nanomaterials. Emphasis is placed on the synthesis of silver and noble metal composites, which demonstrate promising properties for catalytic, antimicrobial, and sensing applications. The influence of synthesis parameters, such as dose, pH, and matrix characteristics, on nanoparticle size and yield is discussed. Emerging applications of these materials in biomedical devices and environmental technologies are presented. While γ-ray synthesis circumvents issues of contamination and scalability inherent in chemical methods, challenges such as accessibility to radiation sources and control over nanoparticle morphology remain. Future research directions are proposed, including the extension of this technique to multimetallic systems, sulfide-based nanocomposites, and hybrid materials.

## 1. Introduction

Silica-based composite materials have garnered significant attention in recent years due to their versatility, tunable properties, and broad applicability across fields such as catalysis, sensing, energy storage, and biomedicine [1,2,3,4,5]. Gamma-ray radiolytic reduction offers a unique approach by leveraging high-energy radiation to induce chemical transformations in the synthesis of silica-based composite materials [6,7,8]. Gamma rays lead to the excitation and ionization of the solvent, forming radical species and electron—hole pairs. These species can initiate radical reactions or act as reducing agents [9,10]. This method facilitates the formation of nanoparticles within silica matrices without the need for surfactants or capping agents, thereby reducing contamination and enabling the production of high-purity materials. The reliance on radiolytic species as reducing agents offers a controllable and reproducible synthesis route.

Despite its potential, the adoption of γ-ray reduction for synthesizing silica-based composites is still in its developmental stages. Research efforts have predominantly focused on elucidating the underlying mechanisms and exploring noble metal systems, such as silver and platinum, deposited on silica matrices [11,12]. However, the broader application of this method to other metals, multicomponent systems, and hybrid materials remains relatively underexplored.

Given the growing demand for advanced materials with tailored properties and the inherent advantages of γ-ray-induced synthesis, a comprehensive review of this method is both timely and necessary. This article aims to provide an overview of the progress made in the field, highlight the challenges and limitations, and propose future directions to advance the application of γ-ray radiolytic reduction in the development of silica-based composites. The use of γ radiation as a radical initiator source for polymeric composites is not discussed in this review.

### 1.1. Mechanism of Radiolytic Reduction on Silica Matrices

Gamma rays can interact with matter through three mechanisms: (i) the photoelectric effect, in which a γ photon is adsorbed by the nucleus; (ii) Compton scattering, in which an electron is ejected from the atom after interaction with the γ photon; and (iii) pair production, in which a high energy γ ray is converted into an electron–positron pair [13]. The photoelectric effect is more likely to occur at lower γ ray energies and for higher atomic numbers, while pair production requires γ rays with energy more than twice the resting mass of an electron. Compton scattering is more likely to occur at intermediate energy levels.

The primary effect of high-energy radiation (such as γ-rays, X-rays, or particle beams) interacting with a solution containing metal ions is the excitation and ionization of the solvent. Rapid follow-up processes, including dissociation of excited states, ion–molecule reactions, and radical–radical recombination, quickly generate molecular and radical species that can react with metal ions. This interaction produces hydrated electrons, hydroxyl and hydrogen radicals, and hydrogen molecules in aqueous solutions [14,15]. Electrons can also be produced through the Compton scattering effect.

H_2_O (γ) → e_s_^−^, OH·, H·, H_2_, H_2_O_2_

Hydrogen radicals (H·) and solvated electrons are strong reducing agents capable of reducing metal ions to metal atoms. Hydroxyl scavenging species are added in order to prevent the re-oxidation of the metal atoms. Isopropanol is typically used as a hydroxyl radical scavenger as it forms 1-hydroxy-ethylmethyl radicals, which can also efficiently reduce metal ions. The metal atoms then aggregate with excess ions and other atoms to form clusters.

(CH_3_)_2_CHOH + OH· (H·) → (CH_3_)_2_C·OH + H_2_O (H_2_)

n e_s_^−^ + M^n+^ → M^0^

n (CH_3_)_2_C·OH + M^n+^→ M^0^ + n (CH_3_)_2_CO + n H^+^

The mechanism of silver nanoparticle formation at the surface of silica materials has already been studied intensively. This is explained by the low reduction potential of Ag^+^, the one-electron reduction reaction, and the obtained Ag NPs being easily quantified through UV–vis spectroscopy [16,17]. Equilibrium is established between the free silver ions in the solution and those adsorbed within the silica’s pores when an aqueous solution of silver ions is added to solid silica.

SiO + Ag^+^ ⇌ [SiO-Ag^+^]

Silver ions can interact with the silanol groups (Si-OH, Si-O^−^) present on the internal pore surfaces of the silica framework. Electrostatic interactions are responsible for Ag^+^ adsorption because silica is negatively charged at a pH higher than ~2. The strong coupling of Ag^+^ ions to the silica structure likely facilitates the scavenging of additional electrons generated within the silica matrix. The radiation energy is absorbed both by the solution and the silica when irradiating aqueous solutions with a high solid silica load. Charge carriers form in the silica cross the solid–liquid interface, with electrons appearing in the aqueous phase, while holes remain trapped within the silica matrix [18].

SiO_2_ (γ) → e _SiO2_^−^ + h _SiO2_^+^

The escaped electrons participate in reducing the silver ions. Silver ions attached to the solid surface (Ag^+^_ads_) or within the mesopores are reduced by radiolytic species originating from both the silica and the surrounding aqueous phase within the silica channels.

e _SiO2_^−^ + Ag_ads_^+^ → Ag_ads_^0^

e^−^_s_ + Ag_ads_^+^ → Ag_ads_^0^

(CH_3_)_2_C·OH + Ag^+^ → Ag^0^ + (CH_3_)_2_CO + H^+^

Hydrated electrons produced in the aqueous phase diffuse to the pore wall and along the silica surface to encounter adsorbed silver ions. Charge transfer from the hydrated electron to the silica is not likely due to the wide band gap of insulating SiO_2_. Pulse radiolysis experiments have shown that the silica surface favors the reduction of adsorbed Ag^+^ ions and stabilizes the Ag_2_^+^ clusters [19]. The stabilization of the Ag_2_^+^ cluster lowers the rate of nanoparticle formation at the silica surface.

Ag^0^_ads_ + Ag^+^ → Ag_2_^+^_ads_

Ag_2_^+^_ads_ + Ag^+^ → Ag_3_^2+^_ads_

Additional insight into the gamma ray reduction mechanism was provided by Miyoshi et al. [20]. The authors used ^18^F as a source of γ-ray. This isotope emits a positron, which reacts with an electron to form two 0.511 MeV γ-rays [21]. The ^18^F isotopes can be directly added to the precursor solution, generating γ-rays in situ, while the 110 min half-life of the isotope ensures that no significant radioactivity is present after a week. Ag nanoparticles or aggregates can be obtained, depending on the experimental conditions. Ag NPs are formed if the silver ions were previously adsorbed onto the silica surface, as the reduction is faster than the adsorption process. The presence of the SiO_2_ NPs was also found to increase the reduction rate of the silver cations.

The γ-ray reduction of Ag^+^ embedded into soda lime silicate glass (74 SiO_2_, 16 Na_2_O, 10 CaO mol.) was studied [22]. AgNO_3_ up to 150 ppm could be dissolved into the melted glass. γ-ray irradiation at low temperatures followed by annealing at 90 °C resulted in the formation of Ag^0^ atoms, while increasing the temperature to 140 °C showed the formation of Ag_2_^+^ species, as evidenced by a 310 nm UV–vis peak associated with the Ag_2_^+^ dimer. Temperatures above 367 °C resulted in the appearance of the 400 nm plasmon resonance peak, indicating metal clusters of at least 13 Ag atoms. The growth of Ag clusters was caused by the diffusion of Ag^0^ and Ag^+^ species. The recombination of positive holes generated during the radiation treatment might have caused the reoxidation of Ag^0^ atoms before cluster formation.

### 1.2. Comparison of Radiolytic Reduction with Other Synthesis Methods

A comparative analysis of γ-ray reduction reactions with the established chemical synthesis methods can highlight the advantages and disadvantages of the radiolytic method. Silver nanoparticles deposited onto silica matrices are a good example due to their versatility and the breadth of literature on their synthesis. Both physical (top-down) and chemical (bottom-up) approaches can be used. The top-down approaches include milling, laser ablation, thermal evaporation, sputtering, and lithographic methods, while the bottom-up strategies consist of chemical methods, such as solution reduction, gas-phase reduction, thermal decomposition, pyrolysis, and chemical vapor deposition or sputtering [23].

Methods such as laser ablation, thermal evaporation, lithography, chemical vapor deposition, and sputtering can be employed to obtain thin films in the case of silver nanomaterials. Milling, chemical methods, and thermal decomposition approaches are useful for obtaining Ag nanoparticles. Milling necessitates large amounts of energy, can lead to crystal phase destruction, and requires stabilizing agents for producing particles smaller than 20 nm, but it is an easily scalable method that does not involve large amounts of solvents [24]. Laser ablation, thermal evaporation, and sputtering can target a solution containing stabilizing agents to obtain Ag nanoparticles less than 30 nm in diameter [25]. These methods require specialized equipment and are difficult to scale up. Thermal decomposition or pyrolysis requires high temperatures and may result in polydisperse nanoparticles. As with the other types of chemical syntheses, these methods require additional reagents and stabilizers [26]. There are various chemical reduction routes for the preparation of Ag NPs, such as the citrate route, polyol method, NaBH_4_ or Na_2_S reduction, and gaseous H_2_ reduction [27]. Chemical methods require solvents and stabilizing agents but give good control over the size and shape of the resulting nanoparticles, as well as being easy to scale up.

The radiolytic reduction method is similar to chemical methods, using the same precursors, but without the need for stabilizing agents. γ-Ray reduction is much slower than chemical methods, thus offering more control over the size of the resulting nanoparticles. The dose and dose rate control the nucleation and growth of the metal particles so that nanoparticles below 5 nm can be readily obtained. Furthermore, the composites obtained by radiolytic means require fewer separation steps than those obtained by chemical methods and they are sterile, which can be an advantage in biological applications.

### 1.3. Types of Silica Matrices

Silica (SiO_2_) is a versatile and widely available material, finding widespread use as a matrix in composite materials due to its exceptional physicochemical properties [28,29]. Its high thermal stability [30,31], chemical inertness [32], and mechanical strength [33] make it an ideal choice for a broad range of applications, from structural components to advanced functional materials [34,35,36]. The porous nature of silica, particularly in its mesoporous and nanoporous forms, provides opportunities for enhancing the mechanical and functional performance of composites through tailored interactions with other chemical components, such as fibers, nanoparticles, or polymers [37,38,39]. Furthermore, silica’s compatibility with surface modification techniques ensures that the surface properties can be adjusted to fit the desired application [40]. Silica-based composites have therefore attracted significant attention in a variety of fields, such as aerospace, energy, biomedical engineering, and environmental technologies [41,42,43].

**Figure 1:**
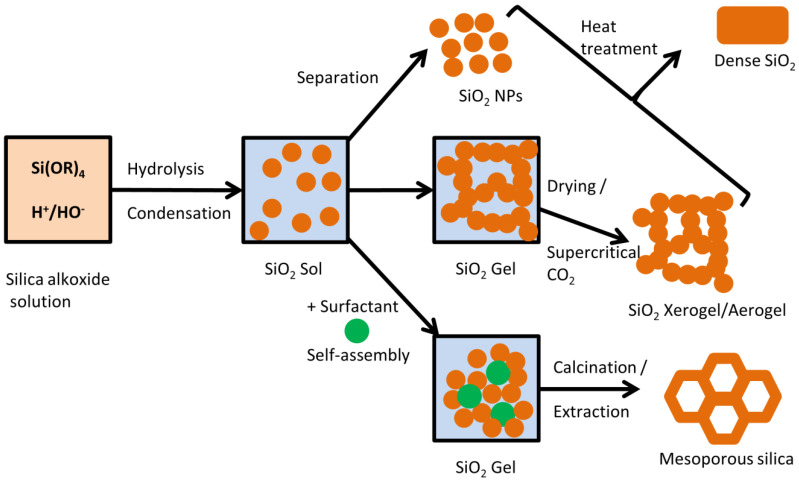
Schematic representation of the silica sol–gel process.

The most common synthesis approach for the various types of silica matrices is the sol–gel method [44]. The sol–gel method consists of the hydrolysis and condensation of a suitable alkoxide precursor (i.e., tetraethyl orthosilicate, TEOS) which results in the formation of a colloidal suspension of nanoparticles (the “sol”). Further condensation reactions lead to the crosslinking of the colloidal particles into a continuous 3D structure (the “gel”). Solvent removal through drying or supercritical CO_2_ extraction leads to the synthesis of xerogels or aerogels, respectively [45]. The introduction of surfactants to the sol-gel process can template pores in the final silica sample, giving rise to mesoporous silica nanomaterials [46]. Silica nanoparticles (NPs) can be obtained directly from the sol. The Stöber process is the most common approach to SiO_2_ NPs since it gives rise to controllable and monodisperse particles in a wide diameter range (Figure 1) [47].

Other synthesis pathways for silica matrices are also possible. Hollow silica microspheres can be prepared by using polymeric particles as hard templates and removing them after the sol–gel synthesis [48]. Porous glass can be obtained from a multi-component dense glass by the acid etching of a soluble glass component [49]. Fumed silica has a structure consisting of 5–50 nm amorphous SiO_2_ nanoparticles fused into a three-dimensional branching structure [50]. Fumed silica is obtained by the pyrolysis of SiCl_4_ and is commercially available under different names, depending on the producer (e.g., Aerosil, Cab-o-sil, etc.).

Silica nanomaterials have a large surface area, with typical values ranging from 100 to 1000 m^2^g^−1^. Pendant silanol groups (Si-OH) are present on the silica surface, with their density depending on the synthesis conditions, such as calcination temperature. The silanol moieties are negatively charged in aqueous media, with pKa values ranging from 2 to 9, depending on their chemical environment [51]. The silica surface is thus negatively charged and can adsorb positively charged species, such as metal cations, at pH values higher than 2.

The pendant silanol groups can be used to react with various organic Si alkoxides. The functionalization of the silica surface through chemically grafted organic groups can be used to tailor the matrix properties, including acidity/basicity, zeta potential, hydrophobicity, or hydrophilicity. Both organic groups and doping elements can be introduced directly to the silica matrix synthesis by using appropriate precursors [52]. These approaches can be used to precisely tailor the surface properties of the silica matrices, offering a wide range of possible properties.

### 1.4. Silica Matrices Obtained Using γ Radiation

The impact of gamma radiation on the structure of silica gels synthesized using tetramethoxysilane (TMOS) as a chemical precursor, with TMOS/H_2_O molar ratios ranging from 1:4 to 1:32, was investigated [53]. Samples were exposed to gamma radiation from a ^60^Co source at doses of up to 200 Gy. The sol–gel process typically begins with a solution containing metal–organic compounds, such as metal alkoxides [54]. The solution transforms into a sol through hydrolysis and polycondensation reactions, where clusters of molecules or fine particles are dispersed in the liquid phase. Continued reaction causes the sol to gel in most cases. Heating the gelled material at higher temperatures changes the gel to a glass, ceramic, or composite [55]. The variation of physical and chemical factors affects the polymerization process and the properties of the final product.

The influence of 180 Gy, 190 Gy, and 200 Gy doses applied after the addition of the Si precursor was investigated in comparison with a non-irradiated sample [53]. The specific surface areas increased with higher water content, as did the pore volumes. This trend was attributed to the enhanced hydrolysis rate at higher water concentrations. The accelerated hydrolysis promoted faster condensation and increased the cross-linking of polymeric chains. The sample porosity changed from microporous to mesoporous when the water content was increased. The irradiated xerogels exhibited larger specific surface areas and pore volumes compared to those prepared via the conventional sol–gel method. Gamma radiation enhanced the overall stabilization of the polymerization process, resulting in more reproducible gel structures. [55]

## 2. Silica Composites Prepared by Radiolytic Reduction

### 2.1. Bulk SiO_2_ and Nanoparticles as Matrices

One of the earliest attempts to obtain composite materials containing silver nanoparticles and silica through radiolytic reduction was reported by Zhu et al. [8]. An aqueous solution containing AgNO_3_, sodium dodecyl sulfate, and isopropanol was subjected to an 8.1–30 kGy dose. The resulting colloidal Ag suspension was added to a silica sol prepared from tetraethyl orthosilicate (TEOS) in an acid medium. Gelation and precipitation occurred by increasing the pH to 8, resulting in composite Ag NPs/SiO_2_ materials. The effect of surfactant concentration and radiation dose was investigated. Increasing the surfactant concentration decreased the Ag NP average diameter from 40 nm to 6 nm, while increasing the dose from 8.1 kGy to 30 kGy increased the Ag NP size from 6 nm to 15 nm.

The synthesis of metallic Ag clusters deposited on fumed silica nanoparticles with an average diameter of 7 nm was investigated by Ramnani et al. [56]. An aqueous silver nitrate solution (0.5 mM) containing 0.2 M isopropanol was used as the metal source. A dose rate of 0.9 kGy obtained using a ^60^Co gamma source was found to completely reduce the silver ions. Increasing the Ag^+^ initial concentration resulted in larger Ag^0^ nanoparticles (NPs), as evidenced by the shifting of the plasmon resonance absorption peak to higher wavelengths. The effect of the initial solution pH on the size of silver nanoparticles was investigated using a higher AgNO_3_ concentration and a lower radiation dose. pH values of 7 and below did not significantly alter the average Ag particle dimensions, while the alkaline medium resulted in higher average diameters and polydisperse particle size distribution. The same authors prepared a sample containing 5% Ag loaded onto silica by using a 60 kGy dose. The dose was chosen to be 25% higher than the theoretical value, which was computed as 5.7·10^−4^ M Ag reduced per 1 kGy [56]. The nanocomposite was recovered by centrifugation. The solution pH decreased from 7 to 2.4 during the gamma radiation reduction of Ag^+^.

**Figure 2:**
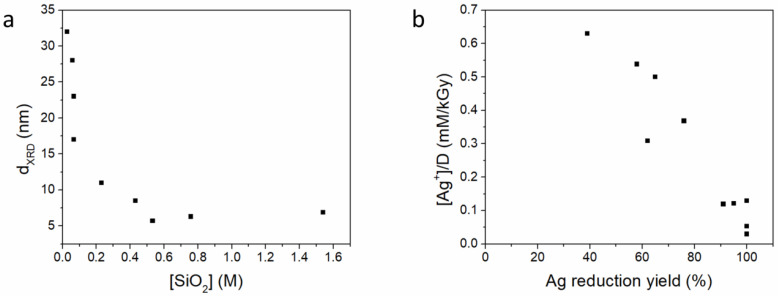
(**a**) Influence of starting silica concentration on silver nanoparticle diameter and (**b**) the relationship between the silver reduction yield and the initial Ag^+^ concentration per kGy radiation dose. Data from [57].

Gamma radiation was also used to prepare Ag–hydrophilic colloidal silica (Aerosil 200) composites [57]. The colloidal silica matrix consisted of particles with an average diameter of 12 nm. The initial SiO_2_, Ag^+^, and isopropanol concentrations were varied, as well as the radiation dose and dose rate. Large Ag aggregates were formed in the absence of silica. Increasing the initial SiO_2_ concentration resulted in smaller particles (Figure 2a). Dispersed 3–6 nm Ag nanoparticles on silica were obtained at high silica concentrations. This formation was attributed to the ionic and stearic stabilization of Ag_n_^z+^ clusters by silica during the radiolytic synthesis. Initial silver ion concentrations of up to 1.3·10^−4^ M per kGy yielded silver reduction yields of over 90%, while higher concentrations resulted in lower yield values (Figure 2b).

**Figure 3:**
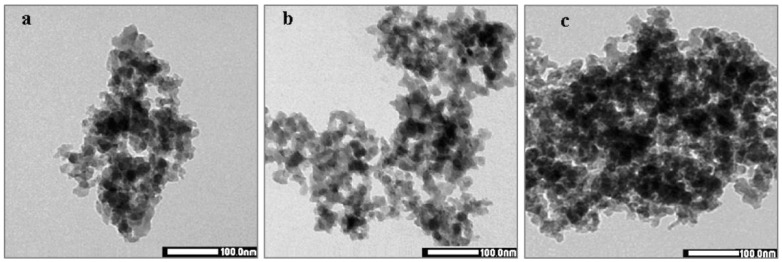
Transmission electron microscopy (TEM) images of Ag/SiO_2_ nanoparticles prepared using initial Ag^+^ concentrations of (**a**) 5 mM, (**b**) 10 mM, and (**c**) 20 mM. Reproduced from [58], under the CC BY 3.0 license.

Ag nanoparticles of 5–40 nm were deposited on 200–300 nm SiO_2_ nanoparticles using an 80:20 (*v*/*v*) water/ethanol solution [58]. Initial silver nitrate concentrations were varied between 5 and 20 mM. The metallic Ag nanoparticle diameter was proportional to the initial AgNO_3_ concentration, increasing from 5–20 nm for the 5 mM Ag^+^ solution to 20–40 nm for the 20 mM Ag^+^ solution (Figure 3). A dose of 1.2 kGy per 1mM Ag^+^ was employed. The composites were blended with high-density polyethylene in order to create plastics with antimicrobial properties at a target of 500 ppm Ag.

SiO_2_ nanoparticles of 12 nm, AgClO_4_, and isopropanol were irradiated with a dose of 10 kGy in aerated, aqueous solutions [59]. Metallic nanoparticles were obtained in the presence of silica nanoparticles. The Ag NPs could also be synthesized in the presence of various species, such as rhodamine, fluorescein, Pb^2+^, or I_2_. Ag NPs did not form in the absence of SiO_2_. The concentration of metallic nanoparticles on the silica matrix was proportional to the radiation dose. Changing the silver ions or silica initial concentrations led to a difference in Ag NP particle size, as evidenced by changes in the plasmon resonance UV–vis peak.

Ni-Pt nanoalloys were deposited onto SiO_2_ particles using radiolytic reduction [12]. Ni is a more electropositive metal than Pt and small Ni^0^ clusters can be spontaneously re-oxidized when exposed to oxygen. Ni-Pt alloy nanoparticles can remove this drawback. The metal salts were adsorbed onto the silica particles as the [Ni(NH_3_)_6_]^2+^ and [Pt(NH_3_)_6_]^2+^ complex cations, as positive species could interact electrostatically with the negatively charged silica surface. Concentrations of 15 mM for Ni^2+^ and up to 10 mM for Pt^2+^ were used, while γ irradiation was carried out with a 250 kGy dose at a dose rate of 10 kGy h^−1^.

**Figure 4:**
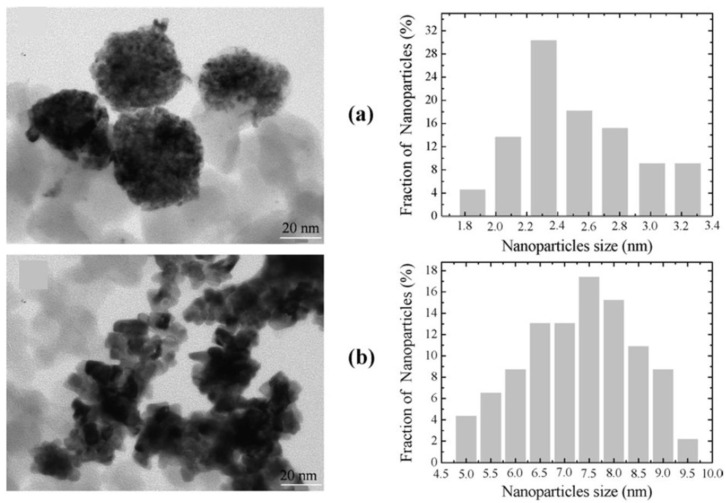
TEM micrographs (**left**) and particle size distribution (**right**) for the (**a**) Ni-15%Pt/SiO_2_ and (**b**) Ni-95%Pt/SiO_2_ samples from [12]. Reprinted from Materials Science and Engineering: B, 177 (1), Benguedouar, Y., Keghouche, N., Belloni, J., Structural and magnetic properties of Ni–Pt nanoalloys supported on silica, 27–33, Copyright (2012), with permission from Elsevier.

The interfacial NiSi phase was noticed in all samples. The intermetallic Ni_3_Pt and NiPt_3_ phases were obtained for 15–35% at. Pt/(Pt + Ni) and 85–95% at. Pt/(Pt + Ni) ratios, respectively. Magnetic measurements showed that the presence of Pt favored the nickel reduction yield and enhanced the magnetization of the Ni–Pt clusters. TEM, X-ray diffraction (XRD), and magnetic measurements were used to conclude that two populations of metallic nanoparticles with diameters of 2–4 nm and ~13 nm were obtained, with the former in higher abundance (Figure 4).

Silica gel particles were functionalized with sulfonic acid through the radiolytic reaction with concentrated sulfuric acid [60]. The highest S/O atomic ratio was obtained for an 80 kGy dose, applied with a dose rate of 1.11 kGy h^−1^. The presence of bound sulfonic acid groups was inferred from various characterization techniques, such as infrared and energy-dispersive X-ray spectroscopy, thermogravimetry, and calorimetry.

A nanocomposite containing CdS NPs deposited onto SiO_2_ nanoparticles was prepared by γ irradiation [61]. Silica nanoparticles with average diameters of 350 nm were obtained through the Stöber process and used as matrices. The semiconductor CdS NPs with average diameters of 20 nm were synthesized in the presence of PVP as a stabilizer. Photoluminescence spectra with an excitation wavelength of 270 nm showed that the CdS/SiO_2_ NPs had an additional emission peak, which was attributed to the band edge emission arising from the direct recombination of the conduction band electrons and the valence band holes [61].

Core–shell particles containing a silica core and ZnS shell were fabricated using γ irradiation and further used to create hollow ZnS microspheres [62]. Monodisperse 560 nm SiO_2_ particles were obtained using the Stöber process. The ZnS shell was synthesized using zinc acetate and thioacetamide as reactants and isopropanol as the oxidative radical scavenger. A dose of 19.5 kGy was employed for the ZnS synthesis. The reaction was monitored by UV–vis spectroscopy. The ZnS peak increased in intensity with reaction time and became blue-shifted, indicating quantum size effects. The formation of the ZnS shell was explained by the adsorption of positive Zn^2+^ ions onto the negatively charged silanol groups presented on the silica surface, prior to the radiolytic reduction of thioacetamide to S^2−^ and the formation of ZnS.

### 2.2. Porous SiO_2_ Matrices Used in the Radiolytic Reduction Reactions

Stable silver nanoparticles immobilized in mesoporous silica were obtained by radiolytic reduction [14]. Two different mesoporous silica with interconnected, wormlike pore morphology were obtained and used as matrices. The mesoporous silica had average pore diameters of approximately 2 nm and 4 nm. Silver ions were adsorbed from a concentrated aqueous Ag_2_SO_4_ solution, in dark conditions. Then, 0.2 M isopropanol was used as a radical scavenger, while oxygen was removed from the reactant solution by purging Ar (Table 1). A high dose rate (7 MGy h^−1^) led to smaller, 2 nm diameter Ag particles, while a lower dose rate (2.7 kGy h^−1^) resulted in the formation of 3–4 nm diameter metal particles in the case of a silica matrix with 4 nm mesopores. The same trend was noticed for the silica with 2 nm mesopores, with 1 nm Ag NPs obtained at 7 MGy h^−1^ and 1.6 nm Ag NPs obtained at 2.7 kGy h^−1^.

**Figure 5:**
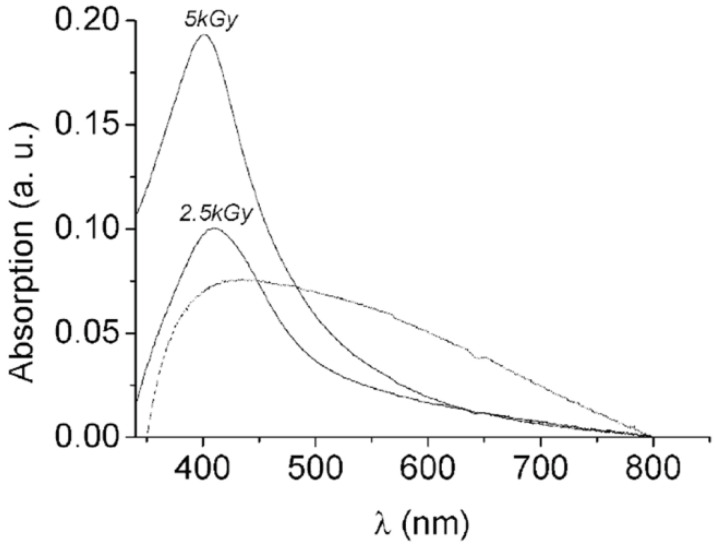
UV–vis spectra of mesoporous silica–Ag NP samples obtained at 2.5 and 5 kGy with a dose rate of 7 MGy h^−1^ (full lines) and 5 kGy with a dose rate of 2.7 kGy h^−1^ (dotted line). Adapted with permission from [14]. Copyright 2003 American Chemical Society.

Ag–silica samples irradiated with doses of up to 4 kGy show a plasmon resonance absorption peak at around 420 nm. This resonance is characteristic of isolated silver nanoparticles and indicates no electronic interaction between individual nanoparticles, despite the high particle density in the mesoporous support. The intensity and shape of the plasmon resonance is influenced by the dielectric environment surrounding the particle. The plasmon resonance maximum for silver particles adsorbed into silica matrices typically appears between 400 and 440 nm [63,64]. The width of the silver particle surface plasmon resonance broadens at higher doses of 5 kGy and lower dose rates (2.7 kGy h^−1^) (Figure 5). The peak position shifts to longer wavelengths (red-shifts), while the composite sample turns from yellow to dark gray. This shift and broadening in the absorption spectrum suggest either electronic interactions between particles or a wide particle size distribution, indicating that the system begins to form an extended metallic structure within the pores. Electron microscopy has further confirmed the effects of particle polydispersity and aggregation. Similar spectral changes occur in silver particles during thermal reduction, where the plasmon band shifts to higher wavelengths as the thermal annealing progresses.

**Figure 6:**
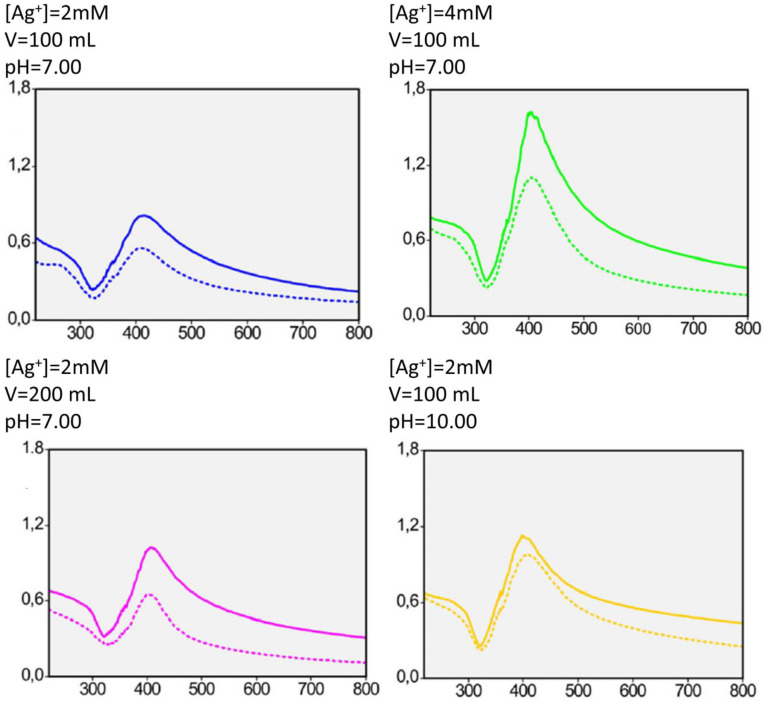
UV–vis analysis of different MCM-14/Ag NP samples obtained using doses of 1 kGy (dotted line) and 4 kGy (full line). Adapted from [11], under the CC-BY license.

The influence of dose, Ag^+^ concentration, volume, and pH on the amount of metallic Ag deposited into MCM-41 mesoporous silica was investigated through factorial design experiments [11]. The final Ag content was found to increase with dose and pH and decrease with solution volume. The initial Ag^+^ concentration did not directly influence the final Ag content. The interaction of the initial Ag^+^ concentration with the other factors was found to be statistically significant as the [Ag^+^]–volume–pH factor increased the final metal content, while the [Ag^+^]–pH decreased it. Using a basic medium (pH = 10) resulted in the formation of silver oxide on the particle surface, even though XRD data only confirmed the presence of metallic Ag. Dose was found to have the most positive effect on metallic Ag yield. The [Ag^+^]–volume–pH factor and the volume had similar effects, even though the former increased the Ag content, while the latter decreased it. The same trend was noticed for pH and the [Ag^+^]–pH interaction, with the former increasing the final silver yield and the latter decreasing it. These two factors had the least effect of all statistically significant factors. The plasmon resonance peak of Ag NPs could be noticed for all samples (Figure 6).

**Figure 7:**
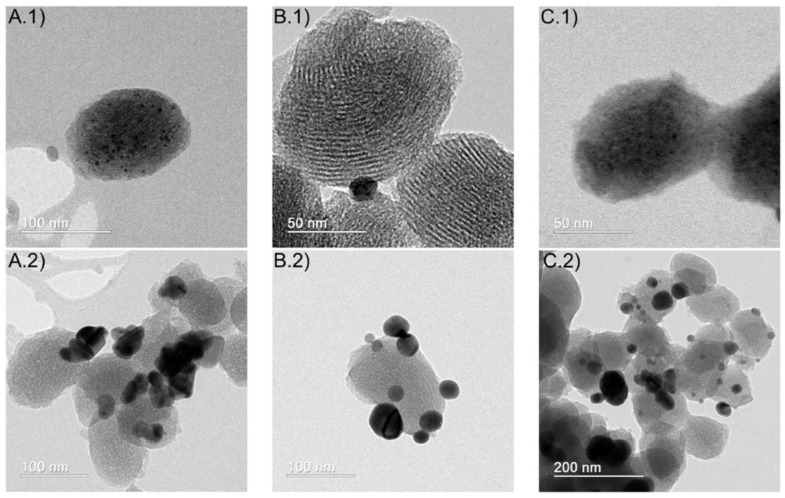
TEM micrographs of MCM-14/Ag NP samples obtained using the following conditions: (**A**) [Ag^+^] = 2mM, pH = 7, dose = 4 kGy; (**B**) [Ag^+^] = 4 mM, pH = 7, dose = 4 kGy; and (**C**) [Ag^+^] = 2 mM, pH = 10, dose = 4 kGy. Images denoted with “1” show Ag NPs, while images denoted with “2” show Ag agglomerations. Adapted from [11], under the CC-BY license.

Physico-chemical characterization of MCM-41/Ag NP samples revealed a decrease in textural properties (specific surface area, average pore diameter, total pore volume) in comparison with pristine MCM-41 silica. TEM measurements confirmed the presence of metallic silver both as nanoparticles inside the mesoporous silica channels and as agglomerations on the external SiO_2_ particle surface (Figure 7). The presence of the silver nanoparticles does not significantly change the zeta potential of the mesoporous silica particles, indicating that there are no strong chemical interactions between the surface silanol groups and the Ag NPs. Biocompatibility assays have indicated that the nanocomposites containing silver do not possess significant toxicity up to concentrations of 200 μg mL^−1^ in comparison with a control [11].

**Figure 8:**
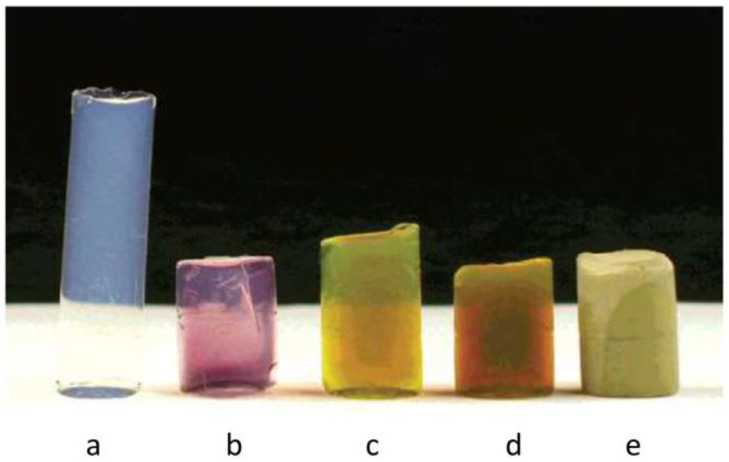
Photographs of (**a**) pristine aerogel, (**b**) aerogel with [Au^3+^] = 10^−4^ M, (**c**) aerogel with [Ag^+^] = 10^−4^ M, (**d**) aerogel with [Ag^+^] = 10^−3^ M, and (**e**) aerogel with [Ag^+^] = 0.18 M. Reprinted with permission from reference [65]. Copyright 2003 American Chemical Society.

Silica aerogel composites containing either Au or Ag were prepared by the gamma irradiation of hydrogel precursors loaded with either AgNO_3_ or HAuCl_4_ solutions containing 0.2 M isopropanol [65,66]. The metal precursor solution concentration was varied between 10^−4^ M and 1 M (Figure 8). In a follow-up study, four successive impregnation/radiation steps were performed for each sample while keeping the experimental conditions the same [65]. A yield of 5.7·10^−4^ M [Ag^+^]/kGy was noticed for dilute solutions. This was equal to the theoretical yield, computed from six reducing electrons per 100 eV. The yield, however, increased to 16 mM [Ag^+^]/kGy when the dose increased to 11 kGy and the initial silver nitrate concentration increased to 10^−2^ M. The authors proposed that radiation-induced chemical reactions were responsible for the increase in yield.

The average particle size increased with increasing metal solution concentration, from 45 nm for 10^−4^ M AgNO_3_ to 170 nm for 0.18 M AgNO_3_. A similar trend was obtained for Au/SiO_2_ composites. The synthesis of composites with high metal loading was performed using a 6 M AgNO_3_ solution and a 400 kGy dose.

**Figure 9:**
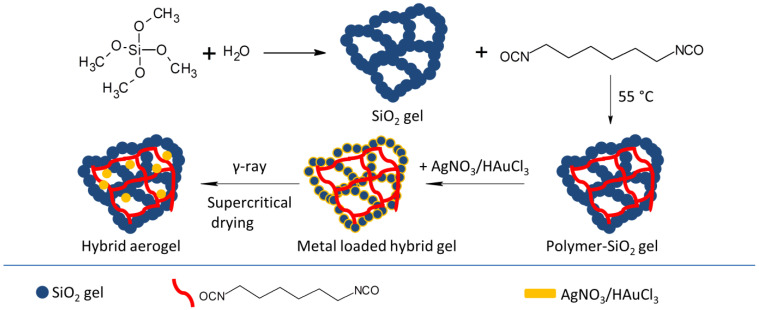
Schematic representation of the synthesis of a hybrid SiO_2_–polyurethane aerogel loaded with Ag or Au NPs [67].

The possibility of creating mechanically strong hybrid organic–inorganic aerogels containing Ag or Au NPs was also investigated [67]. A silica gel was first obtained through the hydrolysis and condensation of tetramethoxysilane in a basic medium, followed by solvent exchange with acetone and hexamethylene diisocyanate. The diisocyanate was polymerized at 55 °C, creating a cross-linked polymer network inside the silica gel. The hybrid material was then impregnated with an acetone solution of Ag^+^ or Au^3+^ containing 0.2 M isopropanol and then subjected to radiolytic reduction at a 7–7.5 kGy dose. The materials were washed with acetone and dried using supercritical CO_2_ in order to obtain the hybrid aerogels (Figure 9). The metal particles had an average diameter of 10.5 nm and good dispersion in the aerogel matrix. The incorporation of the metal nanoparticles did not influence the mechanical properties of the hybrid aerogels as the resulting materials had similar modulus of elasticity and load at rupture values as the aerogel. Thermal stability up to 300 °C was reported for both metal-loaded and unloaded aerogel samples. The radiolytic reduction was interestingly carried out in the absence of water, using acetone as a solvent and isopropanol as a radical scavenger [67].

A mesoporous silica gel with, on average, 3.6 nm pore diameters was used to adsorb Ag^+^ from a 10 mM AgNO_3_ aqueous solution and subjected to a 15.67 kGy radiation dose [68]. No radical scavenger was used. The Ag^0^ content was computed as 1.26% wt., while the nanoparticle diameters were found to be 1.0 nm. The composite was heat-treated and the average Ag NP diameter increased to 2.3 nm for the sample annealed at 650 °C. The authors interestingly noticed changes in the plasmon resonance UV–vis peak due to interactions between the Ag NPs and silica pore walls.

Poly(vinylpyrrolidone) (PVP) chains were grafted onto aminopropyl-functionalized silica particles with 6 nm average pores and Pd nanoparticles were synthesized using the resulting hybrid nanocomposites and the radiolytic reduction [69]. A radical polymerization initiator containing carboxylic acid groups (4,4′-Azobis(4-cyanopentanoic acid)) was first condensed with the aminopropyl groups present on the porous silica surface. PVP chains were then grafted using the reversible addition−fragmentation chain transfer polymerization technique. The number-average molecular weights of the PVP chains varied between 11.9 kDa and 47 kDa, depending on reaction conditions. Pd (II) acetate was used as the metal precursor, while water, ethanol, or an EtOH: H_2_O (40: 60 *v*/*v*) mixture was used as a solvent. The radiolytic reduction was carried out with a ^60^Co source, with a dose rate of 1.51 kGy h^−1^ and a dose of 10 kGy. A dose of 22.2 kGy per mM Pd^2+^ was used in all experiments.

TEM microscopy evidenced individual Pd(0) nanoparticles constrained in the PVP@SiO_2_ matrix. The average metal NP diameter decreased with increasing PVP chain length from 7.8 ± 1.1 nm for 20 kDa PVP chains to 4.6 ± 0.5 nm for 50 kDa chains.

**Figure 10:**
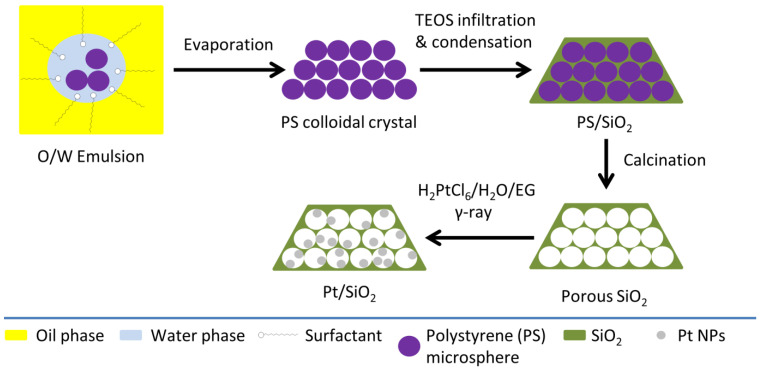
Schematic representation of the synthesis of inverse opal porous SiO_2_–Pt NPs [70].

An inverse opal porous silica matrix was also employed as a matrix for depositing Pt nanoparticles [70]. The inverse opal structure was prepared by using sacrificial polystyrene microspheres (Figure 10). Porous silica with spherical macropores of ~200 nm in diameter was used as a matrix for Pt NPs. A 0.4 mM H_2_PtCl_6_ solution containing 4% vol. ethylene glycol was used for the radiolytic reduction reaction. The dose was varied between 0 and 300 kGy.

**Figure 11:**
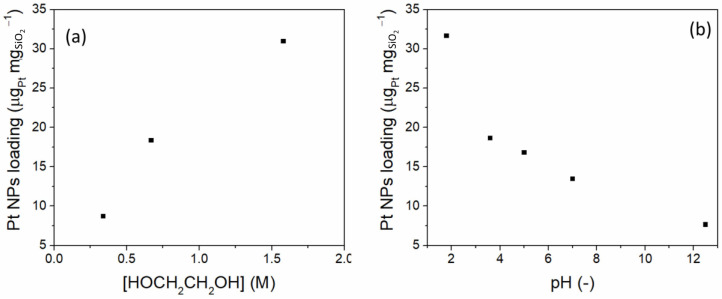
(**a**) Influence of ethylene glycol concentration at a dose of 80 kGy and (**b**) influence of pH on the yield of Pt(0) NPs. Data from [70].

The size of the Pt NPs was found to decrease to 2 nm when the radiation dose increased from 20 to 40 kGy. No metal nanoparticles were noticed at 300 kGy, which was explained by an increase in oxidizing hydroxyl radicals. The size of Pt NPs increased with increasing ethylene glycol concentration and the Pt^4+^ concentration decreased, supporting the reoxidation hypothesis (Figure 11a). The yield of Pt NPs was also found to depend on pH, being higher in an acidic medium (pH = 1.8) than in a less acidic or basic medium (Figure 11b). This observation was correlated with the electrostatic repulsions between the negatively charged silica surface and the [PtCl_6_]^2−^ anions at a pH lower than 3. The optimum conditions for the synthesis of Pt/SiO_2_ were determined to be a dose of 40 kGy, pH = 3.6, and an ethylene glycol concentration of 0.67 M.

SBA-15 mesoporous silica contains hexagonally ordered cylindrical mesopores with average diameters of 6–8 nm. SBA-15 was used as a matrix for the radiolytic reduction of Bi^3+^ [71]. A 0.1 mM aqueous solution of BiOClO_4_ was used as the metal source, while SBA-15 mesoporous silica with an average pore diameter of 6.3 nm and a total pore volume of 0.805 cm^3^g^−1^ was employed as the matrix. The impregnation was carried out under a partial vacuum. The silanol group density of the SBA-15 matrix was changed by varying the calcination temperature between 420 and 570 °C and performing HCl treatments. A larger number of Bi NPs were obtained for the SBA-15 matrix with higher silanol quantity. The matrix treated at 420 °C exhibited dense arrays of 4 nm metal nanoparticles and higher Bi stability under air.

### 2.3. Direct Composite Synthesis

A direct synthesis of silver–silica composites using γ-radiation was also reported [72]. Silver nitrate, sodium silicate, polyvinylpyrrolidone (PVP) as an Ag NP stabilizer, and isopropanol as a hydroxyl radical scavenger were dissolved in water and subjected to gamma ray treatment. A dose of 0.85 kGy per mM Ag^+^ was used. The direct synthesis resulted in ~10 nm Ag^0^ core particles, covered with PVP or silica particles.

A similar approach was used to create an Ag–polyaniline (PANI)–silica nanocomposite material [73]. The PVP stabilizer was replaced with the aniline monomer. A dose of 0.98 kGy per mM Ag^+^ was used in this direct synthesis. The silver radiolysis resulted in spherical metal particles with an average diameter of 30 nm. The UV–vis surface plasmon peak of pure Ag nanoparticles was noticed at 425 nm, while pristine PANI exhibited two UV–vis peaks at 330 and 630 nm. The Ag–PANI–SiO_2_ nanocomposite did not exhibit the Ag surface plasmon peak, while the 630 nm PANI peak had lower intensity, indicating interactions between the metal and conductive polymer. The authors proposed that the aniline monomer was spontaneously oxidized by Ag^+^, forming a silver–polymer core surrounded by a silica shell. The electrical conductivity of the resulting nanocomposite was 223 S cm^−1^, higher than that of pure PANI (21.8 S cm^−1^) or Ag NPs stabilized with PVP (3·10^−8^ S cm^−1^). The surface chemistry of the nanocomposites obtained using PANI or PVP stabilizers indicated different interactions between the silver atoms and the polymers [74].

Nanomaterials containing silica and silver nanoparticles were prepared and incorporated into polypropylene (PP) [75]. The silica nanoparticles were prepared by the hydrolysis and condensation of an aqueous sodium silicate in an acidic medium. Silver was then deposited from a 10:1 H_2_O:iPrOH solution, using sodium dodecylsufate (SDS) as a surfactant. A dose of 25 kGy was used for 15 g of SiO_2_. The final composite materials were obtained by melt blending at 1, 2, and 3% wt. SiO_2_-Ag. The average crystallite size for the Ag NPs was found to be 27 nm from the XRD data, while TEM measurements showed that 30–35 nm SiO_2_ NPs fused with similarly sized Ag NPs. All mechanical properties of the PP composites were improved by the addition of 1% SiO_2_-Ag NPs, but higher mass loading of SiO_2_-Ag decreased the mechanical properties.

Disordered mesoporous silica thin films were obtained by dip-coating and used as a matrix for the radiolytic reduction of Ag^+^ [22]. The films were impregnated with AgNO_3_ aqueous solutions of different concentrations and reduced with a 0.26 kGy γ-ray dose. Ag NPs with diameters varying between 0.9 ± 0.4 nm and 2.7 ± 1.4 nm were obtained by varying the initial Ag^+^ concentration between 0.01 M and 0.1 M. TEM measurements showed that the metal nanoparticles were dispersed into the silica matrix and did not form agglomerations.

**Figure 12:**
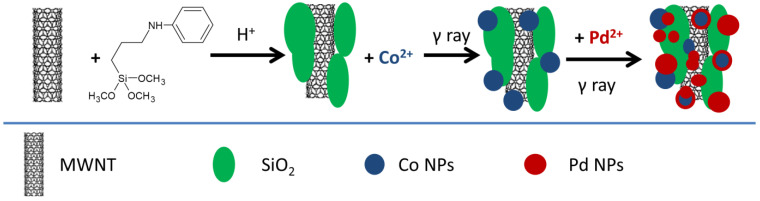
Schematic representation of the synthesis of PdCo NP–MWNT–SiO_2_ nanocomposites [77].

Multi-wall carbon nanotube (MWNT)–silica nanocomposites were also used as matrices for the deposition of metal nanoparticles, such as Pd [76] or PdCo alloys [77]. The carbon nanotubes added electrical and thermal conductivity to the resulting nanocomposites. MWNTs were added to a 7:3 isopropanol/water mixture. A silica source (such as N-[3-(trimethoxysilyl)propyl]aniline), Pd(NO_3_)_2_, and Co(NO_3_)_2_ were then added and the solution was acidified. The reaction mixture was then subjected to γ irradiation with a dose of 30 kGy [77]. The acid medium led to the hydrolysis and co-condensation of the Si precursor. The radiolytic reduction first resulted in the reduction of Co^2+^ to Co^0^. Pd^2+^ was then reduced by both Co^0^ and reducing species from the solvent (e^−^_aq_, H·, etc.), yielding bimetallic nanoparticles (Figure 12). The dose was varied between 10 and 50 kGy, with monometallic nanoparticles being obtained at a dose of 30 kGy. The nanocomposites showed an increased Pd content in comparison with Co. This effect was noticed even for monometallic samples, which contained 3.4 times more Pd than Co. TEM showed the existence of 200 nm SiO_2_ particles decorated with 15–20 nm core–shell metallic nanoparticles. A 5 nm Co core was computed on the basis of the Co/Pd atomic ratio.

**Figure 13:**
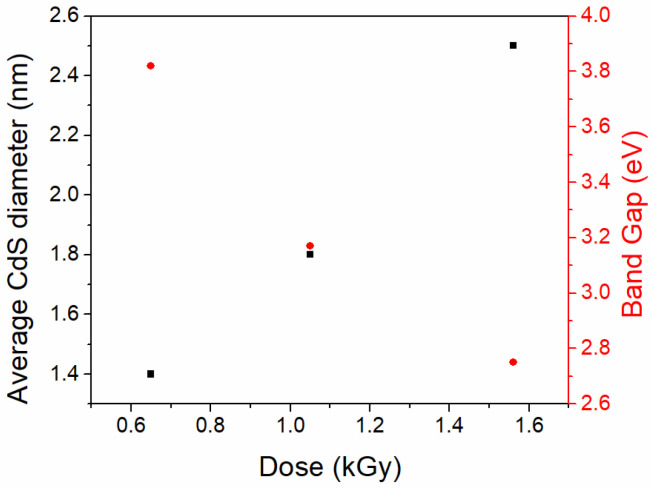
Dose influence on the average CdS particle diameter and band gap. Data from [78].

Semiconductor CdS nanoparticles were also embedded into silica matrices by radiolytic reduction [78]. The silica sol was prepared in the presence of Cadmium (II) acetate and thiourea as the CdS precursors. The reaction mixture was subjected to a 0.65–1.56 kGy dose after the formation of the silica gel. The hydrated electrons formed in water reacted with the thiourea and formed HS^−^ anions, which reacted with the Cd^2+^ cations. The CdS nanoparticles embedded into the silica matrix had average particle diameters between 2 and 5 nm and a hexagonal lattice structure. The semiconductor particle increased with the increasing dose, while the band gap decreased (Figure 13). CdS NPs obtained without the silica gel had a metastable, cubic structure. The hexagonal stable structure of the embedded nanoparticles was explained by the annealing of the sample due to the heat generated inside the silica matrix during the γ ray treatment. Optical absorbance spectra showed a blue-shift of the embedded CdS nanoparticles with respect to bulk, indicating a larger band gap. The deviations from expected band gap values were explained by electrostatic interactions between the CdS NPs and the negatively charged silica surface.

Hybrid materials containing mixed SiO_2_–ZrO_2_ oxides and polydimethylsiloxane (PDMS) as the organic precursor were obtained using doses of up to 700 kGy [79]. Atomic ratios of 0–10% Si–Zr were investigated. The required gamma dose to reach the gelation point was found to depend on the PDMS-to-inorganic precursor volume ratio, increasing as the ratio decreased. Samples with a PDMS/(Si + Zr) ratio of 2:3 were irradiated to doses exceeding the gel point. The gelation dose increased as the Zr precursor content rose. The resulting materials were homogeneous, transparent, and flexible, exhibiting swelling in suitable polymer solvents. The hybrid materials were comprised of a network of dense oxide clusters interconnected by polymer chains. The inclusion of Zr during synthesis led to smaller but denser clusters in the obtained samples.

The synthesis conditions for the samples obtained through the γ-ray reduction method are summarized in Table 1.

**Table 1:** Radiolytic reduction synthesis conditions for various composite materials.

SiO_2_ Matrix	Synthesis Precursor	Dose (kGy)	Reaction Conditions	Reference
Fumed silica (7 nm)	AgNO_3_ (0.5 mM)/H_2_O/iPrOH (0.2 M)	0.9–60	pH 2–9	[56]
Silica aerogel	AgNO_3_/H_2_O/iPrOH (0.2 M) HAuCl_4_/H_2_O/iPrOH (0.2 M)	3.5–400	[Ag^+^] = 10^−4^–6 M [Au^3+^] = 10^−4^–10^−3^ M	[65]
Silica–polyurethane aerogel	AgNO_3_/Acetone/iPrOH (0.2 M) HAuCl_4_/Acetone/iPrOH (0.2 M)	7–7.5	[Ag^+^], [Au^3+^] = 3·10^−4^ M	[67]
Fumed silica (12 nm)	AgNO_3_/H_2_O/iPrOH	9–13	pH = 6.1	[57]
Silica NPs (200–300 nm)	Ag^+^/H_2_O/EtOH (80/20)	2–50	SiO_2_/H_2_O/EtOH = 9:80:20 (*w*/*v*/*v*)	[58]
Na_2_SiO_3_	AgNO_3_/PVP/H_2_O/iPrOH	25	Na_2_SiO_3_/AgNO_3_/PVP = 1:1:1 (*w*/*w*/*w*) 0.85 kGy per mM Ag^+^	[72]
Na_2_SiO_3_	AgNO_3_/ANI/H_2_O/iPrOH	30	Na_2_SiO_3_/AgNO_3_/ANI = 1:1:1 (*w*/*w*/*v*) 0.98 kGy per mM Ag^+^	[73]
SiO_2_ NP	AgNO_3_/SDS/H_2_O/iPrOH	25	H_2_O:iPrOH/SDS = 90:10:1 (*v*/*v*/*w*)	[75]
Mesoporous silica thin film	AgNO_3_/H_2_O	0.26	[Ag^+^] = 0.01–0.1 M	[22]
Mesoporous silica	Ag_2_SO_4_/H_2_O/iPrOH	0.5–5	pH = 7	[14]
MCM-41	AgNO_3_/H_2_O	1; 4	[Ag^+^] = 2; 4 mM pH = 7; 10	[11]
PVP@ mesoporous silica	Pd(OAc)_2_/EtOH, H_2_O, EtOH:H_2_O	10	[Pd^2+^] = 0.45 mM	[69]
SiO_2_ particles	Ni(HCOO)_2_/[Pt(NH_4_)_4_]Cl_2_/ NH_4_COOH/H_2_O	250	[Ni^2+^] = 15 mM [Pt^2+^] = 0–10 mM	[12]
Inverse opal SiO_2_	H_2_PtCl_6_/H_2_O/HOCH_2_CH_2_OH	0–300	[Pt^2+^] = 0.4 mM H_2_O/HOCH_2_CH_2_OH = 96:4 (*v*/*v*) pH = 1.8–12.5	[70]
SiO_2_ NPs (12 nm)	AgNO_3_/H_2_O/iPrOH	2–10	[Ag^+^] = 0.84 mM	[59]
SBA-15	BiOClO_4_/H_2_O		[BiOClO_4_] = 0.1 mM	[61]
MWNT/SiO_2_	Pd(NO_3_)_2_/Co(NO_3_)_2_/iPrOH:H_2_O 7:3	10-50	[Pd^2+^] = 4.7 mM [Co^2+^] = 4.7 mM	[77]
SiO_2_ gel	H_2_O/iPrOH/HNO_3_/Cd(CH_3_COO)_2_/SC(NH_2_)_2_	0.6–1.5	[Cd^2+^] = 56 mM [SC(NH_2_)_2_] = 84 mM	[78]
SiO_2_ particles	Zn(CH_3_COO)_2_/CH_3_CSNH_2_/iPrOH (10% *v*/*v*):H_2_O	19.5	[Zn^2+^] = 24.5 mM [CH_3_CSNH_2_] = 30 mM	[62]

## 3. Applications of Silica Composites Obtained by γ Ray Reduction

### 3.1. Catalysis

Ag nanoparticles of 3–6 nm deposited on, on average, 12 nm particle diameter fumed silica were investigated as catalysts for styrene oxide reduction with 10% gaseous H_2_ [57]. Conversions of up to 16.7% were achieved for the samples containing 5–6 nm Ag nanoparticles. The main reaction product was vinylbenzene, with a selectivity of up to 75%.

Pd NPs of 3–8 nm decorating a PVP functionalized mesoporous silica matrix were tested as catalysts for the hydrogenation of 4-nitrophenol to 4-aminophenol [69]. The effect of the polymer chain length was studied. The larger, 50 kDa PVP chain exhibited lower catalytic activity reduction after multiple reaction cycles than a smaller 20 kDa chain. The composites with the larger PVP chains retained 81.1% of their initial activity, while the materials with the 20 kDa chains had 74.5% of their initial catalytic activity.

Pt nanoparticles of 1.3 nm supported on an inverse opal porous silica matrix with 200 nm average pore diameters were investigated in the *o*-nitroaniline reduction reaction to 1,2-benzenediamine by NaBH_4_ at room temperature [70]. The Pt-containing composite showed catalytic activity in the *o*-nitroaniline reduction and good reusability after five reaction cycles.

### 3.2. Biomedical Applications

**Figure 14:**
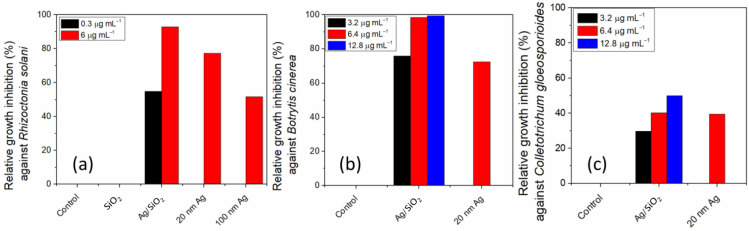
Relative growth inhibition of three fungi by an Ag/SiO_2_ nanocomposite obtained through direct synthesis in comparison with commercial Ag NPs. Data from [72].

Ag^0^ NPs of 10 nm covered with silica spheres of similar dimensions were prepared by direct synthesis and their antifungal properties were evaluated [72]. The nanocomposite showed higher inhibition of *Rhizoctonia solani* and *Botrytis cinerea* than either 20 nm or 100 nm Ag NPs without silica. Similar inhibition was noticed for *Colletotrichum gloeosporioides* for both the 20 nm pristine Ag NPs and the nanocomposite. The fungal inhibition was relatively constant for up to 24 months. The nanocomposite was shown to damage the fungal cell through structured cell membrane destruction. The authors proposed that the silica outer layer was responsible for the slow release of the Ag NPs, which damaged the cell wall (Figure 14).

The anti-microbial activity of the Ag^0^ nanocomposite was also tested using the model plant *Arabidopsis thaliana* [80]. The influence of pristine Ag NPs and the nanocomposite on the plant growth at 4 weeks was tested by spraying the model plants. Concentrations lower than 50 ppm did not result in morphological changes. An increase in plant root growth was noticed for the nanocomposite treated with a 1 ppm Ag suspension. The expression of pathogenesis-related genes implicated in systemic acquired resistance was increased after treatment with the 10 ppm suspension of the Ag nanocomposite. The treated plants had increased resistance towards the *Pseudomonas syringae* pv. *tomato* bacterial pathogen.

**Figure 15:**
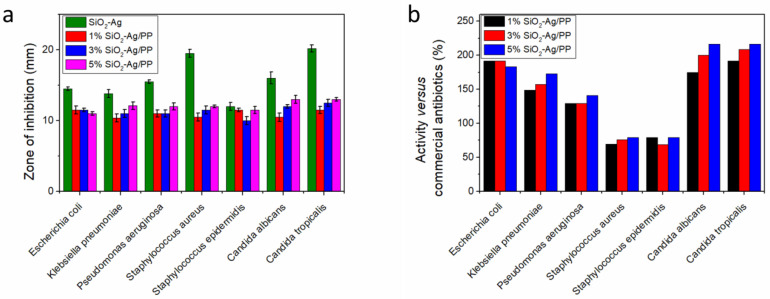
(**a**) Zone of inhibition of SiO_2_–Ag NPs and composites with PP against various bacteria and fungi and (**b**) their corresponding activity in comparison with commercial antibiotics. Data from [75].

The antibacterial and antifungal activity of SiO_2_–Ag nanoparticles and composite materials containing a polypropylene matrix were investigated through the disk diffusion method [75]. Two strains of Gram-positive bacteria (*Staphylococcus aureus* and *Staphylococcus epidermidis*), three strains of Gram-negative bacteria (*Escherichia coli*, *Klebsiella pneumoniae*, and *Pseudomonas aeruginosa*), and two unicellular fungi (*Candida albicans* and *Candida tropicalis*) were employed (Figure 15a). The zone of inhibition diameters were compared with commercial antibiotics and the activities of the composites were computed as the percentage of the two inhibition zones (Figure 15b). The pristine SiO_2_–Ag NPs exhibited higher activity than the PP composites, especially towards *P. aeruginosa*, *C. albicans*, and *C. tropicalis*. The polymer composites still exhibited high antimicrobial activity, making them suitable for use in medical and food packaging applications.

### 3.3. Sensing Applications

A ternary composite containing Ag NPs, polyaniline (PANI), and silica was obtained through the direct synthesis method and investigated as an electrode material for biosensors [81]. The biosensor was fabricated by spin-coating the Ag–PANI–SiO_2_ nanocomposite solution onto a conductive indium tin oxide (ITO) substrate. The biosensor was tested in the H_2_O_2_ reduction reaction, showing a reduction peak at −0.63 V versus the Ag^0^/Ag^+^/KCl(saturated) electrode.

A multiwall carbon nanotube/silica matrix was used as a matrix for Pd, Co, and PdCo NPs [77]. The activity of the resulting nanocomposite was tested in the Oxygen Reduction Reaction (ORR). The peak corresponding to ORR was found at +0.48 V. The bimetallic catalyst had the highest current response for ORR (20.1 μA), which was explained by an increased surface area due to a smaller particle size than the monometallic samples.

## 4. Conclusions

The use of γ rays in the synthesis of silica-based materials through radiolytic reduction reactions is a promising method. Irradiation with γ rays creates free electrons and positive holes in the solvent and silica matrix as well as various radical species. Water is the most commonly used solvent for this type of reaction. Different organic species can be added to aqueous solutions in order to scavenge the oxidizing radicals formed by γ radiation (HO·, etc.) [14]. Isopropanol (2-propanol) was the radical scavenger employed in most previous literature reports. The radicals formed by the reaction of isopropanol with hydroxyl radicals are also reducing agents. Isopropanol is typically added to the reaction media at a 0.2 M concentration.

The reduction of Ag^+^ onto SiO_2_ substrates is the most common use of γ ray synthesis of silica composites. This reaction was also used extensively to study the mechanism of the radiolytic reduction process. Silver reduction is a one-electron process. The formation of metal nanoparticles can happen both at the silica surface and in solution. The γ ray reduction yield is higher at the silica surface [57]. This fact can be attributed to the adsorption of metal ions onto the silica surface. The silanol groups decorating the SiO_2_ matrix are negatively charged at a pH higher than 2, leading to strong electrostatic interactions with the metal cations. Another factor affecting the growth of metal nanoparticles at the interface with the silica substrate is the fact that γ rays can be adsorbed by the inorganic material, leading to electron–hole pairs. The electrons can reduce the adsorbed metal ions. Smaller metal nanoparticles are obtained at the silica–metal interface than in solution. The formation of nanoparticles in solution requires larger doses and results in the formation of agglomerations [8].

Various synthesis parameters influence the size and yield of the final Ag nanoparticles. These include the silver cation initial concentration, dose, dose rate, volume, pH, presence of radical scavengers, and silica surface properties (specific surface area, zeta potential). The radiation dose is proportional to the yield of reduced metal. A yield of 5.7·10^−4^ M [Ag^+^]/kGy was noticed for dilute solutions ([Ag^+^] < 1 mM), which was equal to the theoretical yield expected from six reducing electrons per 100 eV energy [56]. A higher yield per kGy was noticed for higher initial silver concentrations.

The dose rate influences the number of seeds for nanoparticle growth. A higher dose rate leads to the formation of more metal clusters than a lower dose rate [20]. Higher dose rates thus yield smaller nanoparticles. Increasing the initial metal concentration or the radiation dose leads to larger metal nanoparticles as more metal is reduced while keeping the number of seed clusters constant [66].

Interestingly, the solution volume was found to be inversely correlated with the reduced metal yield [11]. This fact supports the hypothesis that more metal is reduced at the interface with silica than in solution as the reducing electrons need a smaller path to encounter the cations. Silica matrixes with a higher surface area or higher silanol density can absorb more metal cations and will therefore increase the reduction yield. A basic pH (i.e., 10) was found to increase the amount of Ag deposited onto silica [11]. This fact was explained by the precipitation of Ag_2_O in basic media.

Other metal–silica composites were reported. Noble metals (Pt, Pd, Au) could also be deposited onto silica substrates using γ radiation [65,69,70]. Bismuth, nickel, and cobalt could also be reduced by γ rays [12,71]. Radiolytic reduction was also employed to obtain bimetallic nanoparticles [77]. The difference in metal reduction potential was used to create core–shell structures as the more electropositive metal could reduce the noble metal in situ. Sulfide-based silica composites were also reported. ZnS or CdS could be obtained through the γ ray reduction of suitable S-containing compounds (thioacetamide, thiourea) [61,62,78]. The radiolytic reduction of metal species in the presence of silica matrices can be used to create multicomponent nanocomposite materials. Polymers or carbon nanotubes could be incorporated using this method, with the aim of increasing the conductivity of the samples.

The synthesis of silica-based materials through γ radiation possesses specific advantages and disadvantages in comparison with traditional chemical or electrochemical methods. The largest disadvantage of γ ray synthesis is the regulatory barriers to suitable γ ray sources. The chemical synthesis of metal nanoparticles is also very fast, while the γ radiation process is carried out over hours or days, depending on the total dose. Chemical synthesis, however, requires stabilizing agents for the obtained metal nanoparticles. γ radiation synthesis can be employed without the need for such agents. Another advantage of the use of γ rays is that this method can be used to obtain small (1–5 nm) nanoparticles deposited onto porous silica matrices, such as xerogels and aerogels or mesoporous silica. Chemical synthesis processes are limited by the diffusion of reducing agents into nanometer-size pores and, consequently, do not produce homogenous nanocomposites. Of special importance for biological applications is the fact γ ray synthesis can result in sterile nanomaterials, without requiring further processing.

The composite materials obtained using γ radiation can be used in a variety of applications. Catalysis, antimicrobial applications, and sensors have been investigated to date. The research performed on the synthesis of silica composites through γ irradiation has been focused more on theoretical aspects than the applicative part so far.

## 5. Perspectives and Outlook

The synthesis of silica-based composite materials through γ-ray radiolytic reduction represents a versatile and promising strategy within the broader field of material science. The method’s ability to generate nanostructured materials without surfactants or capping agents makes it particularly attractive for applications requiring minimal contamination, such as biomedical devices or environmentally sensitive catalysts. While significant advances have been made, there are still opportunities for expanding and refining this field.

The majority of the research so far has focused on noble metals, such as silver, gold, and platinum, deposited onto silica matrices. These studies have provided valuable insights into the radiolytic reduction mechanisms and the effects of synthesis parameters. However, extending this approach to more electropositive metals, such as transition metals or their alloys, could open up new avenues for catalysis and electronic applications. The exploration of multimetallic nanoparticles and core–shell structures within silica matrices is particularly underexplored and could provide access to materials with tunable properties.

The use of γ-ray reduction for synthesizing sulfide-based nanocomposites, such as ZnS or CdS on silica supports, remains in its infancy. Expanding research into such systems could enhance their applicability in optoelectronics and photocatalysis. Furthermore, hybrid materials combining silica with organic polymers, carbon nanotubes, or other nanostructures hold promise for advanced multifunctional materials with tailored mechanical, electrical, or optical properties.

One of the critical challenges in this field is achieving precise control over the size, distribution, and morphology of nanoparticles within the silica matrix. Innovations in radiolytic dose delivery systems, such as pulsed or gradient dose irradiation, could provide more refined control over nanoparticle formation. Additionally, exploring alternative radical scavengers or stabilizing agents could further enhance the reproducibility and uniformity of the synthesized materials.

Investigations into the role of silica surface properties, such as pore size, silanol group density, and surface charge, also merit further attention. Modulating these parameters through tailored matrix preparation methods could significantly influence nanoparticle loading, size, and distribution. Factorial experimental designs and computational modeling can aid in identifying the optimal synthesis conditions. The versatility provided by the silica matrices in terms of chemically adjusting their properties through functionalization with organic groups or doping the SiO_2_ framework with ad-atoms has also not been investigated so far.

While the antimicrobial, catalytic, and sensing applications of silica-based composites synthesized via γ-rays are well documented, the full potential of these materials remains untapped. For instance, the development of biocompatible and sterile materials for targeted drug delivery or tissue engineering could benefit from the inherent sterility of γ-ray-processed composites. Similarly, these materials could be optimized for environmental applications, such as pollutant degradation or heavy metal sequestration.

The combination of metallic nanoparticles with mesoporous or inverse opal silica structures also holds promise for advanced optical devices, including photonic crystals and plasmonic sensors. Further exploration of the electronic and magnetic properties of these composites could lead to breakthroughs in energy storage, electronic devices, or magnetic resonance imaging contrast agents.

The widespread adoption of γ-ray reduction is limited by the availability and regulatory constraints associated with γ-ray sources despite its advantages. Developing alternative radiation sources, such as high-energy electron beams or X-rays, could improve access to this synthesis method. Addressing the scalability of the process is crucial for industrial applications. Future efforts on adapting this technology for large-scale production merit attention.

The synthesis of silica-based composites through γ-ray radiolytic reduction is a field with vast potential for growth. The potential of these materials could be unlocked by broadening the range of materials, refining synthesis techniques, and diversifying their applications. Bridging the current gaps between fundamental research and practical applications will require interdisciplinary collaboration, combining expertise in radiation chemistry, materials science, and engineering. As advancements continue, this method is poised to contribute significantly to the development of next-generation nanocomposite materials.

## Data Availability

Data sharing is not applicable.
